# Natural Compounds Derived from Chilean Species and Their Cytotoxic Potential Against Cancer

**DOI:** 10.3390/cancers18040656

**Published:** 2026-02-17

**Authors:** Tania Koning, Gloria M. Calaf

**Affiliations:** Instituto de Alta Investigación, Universidad de Tarapacá, Arica 1000000, Chile; tkoning@academicos.uta.cl

**Keywords:** cancer, natural compounds, bioactive molecule, Chilean plants, cytotoxic compounds

## Abstract

Cancer remains a pressing global health concern with profound social and economic implications, accounting for nearly 17% of all deaths worldwide. Throughout history, medicinal plants have been employed in traditional healing practices to treat various illnesses, including cancer, and they continue to play a vital role in modern healthcare. Chile, a South American country with a diverse range of climates and landscapes, harbors exceptional biodiversity that offers many natural products with potential cytotoxic properties. This study presents a comprehensive review of the literature, collecting existing evidence that twelve Chilean species, *Leptocarpha rivularis*, *Peumus boldus*, *Aristotelia chilensis*, *Drimys winteri*, *Solidago chilensis*, *Buddleja globosa*, *Senecio graveolens*, *Geoffroea decorticans*, *Ugni molinae*, *Austrocedrus chilensis*, *Gracilaria chilensis*, and *Kageneckia oblonga*, exhibit potential cytotoxic properties against cancer. Extracts and isolated molecules from these species have been shown to induce cell death, reduce cellular viability, attenuate clonogenic growth, induce senescent phenotype, and even exert anti-angiogenic effects. These species display high toxicity and specificity, operating through mechanisms such as cell cycle arrest, induction of apoptosis, chromatin fragmentation, and mitochondrial alteration. The findings summarized here support the importance of further research. Many of the bioactive compounds identified remain poorly characterized, and their molecular mechanisms of action are not yet fully understood. By consolidating current evidence, this work establishes a foundation for future research aimed at developing therapeutic applications that harness Chilean natural products to prevent cancer progression.

## Simple Summary

Medicinal plants have been used in therapy since ancient times and remain important in human health. Traditionally, they have been employed to treat various diseases, including cancer, a major global health challenge with profound social and economic implications, responsible for approximately 17% of worldwide mortality. Numerous studies have identified plant compounds and their mechanisms of action, supporting further clinical research into their therapeutic potential. Within this context, Chile has remarkable biodiversity, characterized by high endemism across varied environments and climates, presenting a valuable source of bioactive compounds that merits deeper exploration. This work gathers and analyzes evidence regarding the cytotoxic properties of twelve Chilean species. These species have demonstrated the capacity to induce cell death, reduce cellular viability, attenuate clonogenic growth, induce senescent phenotype, and even show anti-angiogenic effects. These species display low toxicity and high specificity, operating through mechanisms such as cell cycle arrest, apoptosis induction, chromatin fragmentation, and mitochondrial alteration.

## 1. Introduction

Cancer remains one of the foremost global health challenges, responsible for nearly one in six deaths (16.8%) worldwide and a major contributor to premature mortality (22.8%) from non-communicable diseases [1]. Beyond its impact on life expectancy, cancer imposes substantial social and economic burdens, varying across regions, cancer types, and populations [2].

Based on the projected changes in population growth and aging, and assuming that overall cancer rates remain unchanged, we predict over 35 million new cancer cases (including non-melanoma skin cancers, except basal cell carcinoma) will occur by 2050. This represents a 77% increase compared to the estimated 20 million cases in 2022 [1].

The overall scale of cancer and the diversity of cancer profiles by world region and human development level reemphasize the need for a global escalation of targeted cancer control measures. Investments in prevention, including targeting key risk factors for cancer such as smoking, overweight, obesity, and infections, could avert millions of future cancer diagnoses and save many lives worldwide. Such measures would yield substantial economic and societal benefits for countries worldwide in the coming decades [1].

Plant compounds, naturally abundant, have long intrigued researchers due to their potential anticancer properties. The World Health Organization (WHO) defines medicinal herbs as “any plant that, in one or more of its parts (leaves, flowers, bark, root, etc.), contains substances that make it useful for improving human or animal health, and which can be used for therapeutic purposes or as precursors for semi-synthetic drugs” [3]. The use of medicinal plants for therapeutic purposes is an ancient practice rooted in traditional knowledge, transmitted across generations, and remains a widespread component of human healthcare.

Phytochemical compounds, for example, polyphenols (including flavonoids) and alkaloids found in many plant species, have exhibited anticancer activity [4]. In vitro investigations enable us to understand the cellular mechanisms of action of these compounds, aiding in the selection of promising candidates for further clinical research [5].

High levels of endemism characterize the biodiversity of Chile, which is expressed across diverse environments and under a wide range of climatic and geographical conditions, ranging from the most inhospitable regions for human life to areas abundant in natural resources. Chile, situated at the southwestern tip of South America, spans approximately 4300 km in a north–south direction, extending to nearly 8000 km when the Chilean Antarctic Territory is included. The territorial extension of Chile contributes to its remarkable biological diversity. Nationally, nearly 25% of the described species are endemic, underscoring the uniqueness of the natural heritage of Chile [6]. This work describes and analyzes the natural compounds endemic to Chile that exhibit potential cytotoxic properties against cancer. The work aimed to create a basis for future investigations, enabling an ongoing characterization of the molecular composition of these species, their effects on various cancer types, and potential clinical applications.

## 2. Data Collection Method

**Figure 1:**
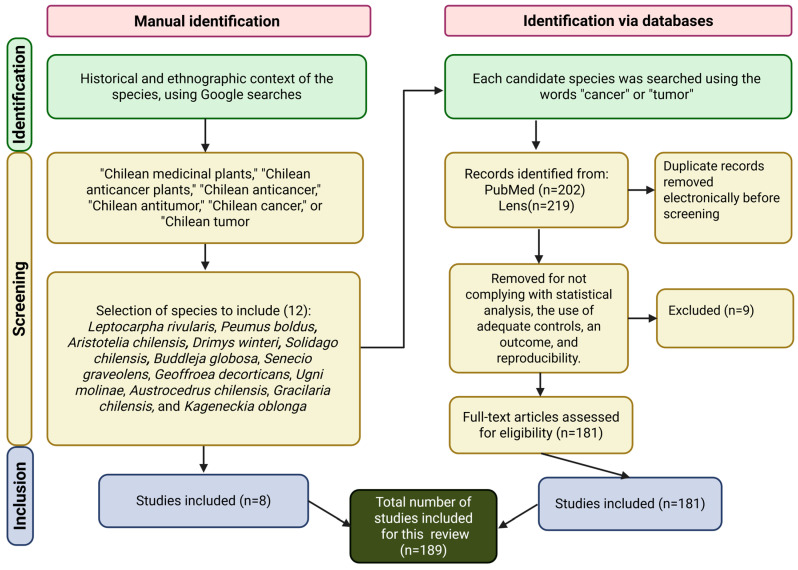
Workflow for the identification of Chilean cytotoxic plant species. The review was conducted between January and October 2025 using PubMed (https://pubmed.ncbi.nlm.nih.gov/ accesses from 1 January to 31 October 2025)) and Lens (https://www.lens.org/ accesses from 1 January to 31 October 2025)), with preliminary searches in Google (https://www.google.com/ accessing from 1 to 15 January 2025) to capture historical and ethnographic context. Search terms included “Chilean medicinal plants,” “Chilean anticancer plants,” “Chilean antitumor,” “Chilean cancer,” and “Chilean tumor.” Candidate species were further screened with the keywords “cancer” or “tumor,” and only studies meeting inclusion criteria (statistical analyses, appropriate controls, defined outcomes, reproducibility) were considered (n = 189).

This manuscript presents an exhaustive review, which examines the literature about Chilean cytotoxic plant species found in journals that are indexed in Clarivate Web of Science^TM^ (WoS), using search engines such as PubMed (https://pubmed.ncbi.nlm.nih.gov/ accesses from 1 January to 31 October 2025) and Lens (https://www.lens.org/ accesses from 1 January to 31 October 2025). Initial queries were performed using Google (https://www.google.com/ accessing from 1 January to 15 January 2025), focusing on the historical and ethnographic context of the species, and the search terms included “Chilean medicinal plants,” “Chilean anticancer plants,” “Chilean anticancer,” “Chilean antitumor,” “Chilean cancer,” or “Chilean tumor.” Candidate species identified through this preliminary search were subsequently investigated in PubMed and Lens, using the keywords “cancer” or “tumor” to retrieve relevant articles and books. Each source was critically analyzed to construct the content of this review. Inclusion criteria required the presence of statistical analyses, appropriate controls, defined outcomes, and reproducibility. Searches were performed in both Spanish and English. The data collection approach is shown in Figure 1.

## 3. Chilean Species and Their Cytotoxic Potential Against Cancer

The extraordinary biodiversity in Chile, shaped by diverse climates, altitudes, and the interaction between marine and terrestrial ecosystems, has prompted species to develop various adaptation mechanisms. The production of secondary metabolites has evolved as a defense strategy against extreme conditions such as ultraviolet radiation, high salinity, low temperatures, or hypoxia at high altitudes [7]. These metabolites, produced in response to environmental factors, fulfill several ecological functions, including protection against herbivores, microbes, and competing plants, as well as signaling to attract pollinators or seed-dispersing [8]. These compounds possess biochemical properties that may be highly relevant for cancer prevention and therapy. In this context, Chilean endemic species represent a unique reservoir of molecules with cytotoxic potential, capable of modulating key cellular processes including proliferation, apoptosis, senescence, and angiogenesis. The following section presents twelve selected species, each discussed individually, whose extracts and isolated compounds have demonstrated anticancer activity in both in vitro and in vivo studies.

### 3.1. Leptocarpha rivularis

*Leptocarpha rivularis* (“Palo negro”) is a native plant that mainly grows in southern Chile. It has been used by the Mapuche people to alleviate gastrointestinal disorders [9]. Infusions prepared from *L. rivularis* have been reported to exert antioxidant and cholinesterase inhibitory activities, attributed to their high content of phenolic compounds [10].

*L. rivularis* is rich in bioactive molecules, including sesquiterpenes, flavonoids, oxylipins, organic acids, phenolic acids, and a group of molecules termed sesquiterpene lactones (SLs) [10,11,12]. SLs have attracted the attention of researchers due to their broad biological activities, encompassing anticancer, antifungal, anti-inflammatory, antimicrobial, and antioxidant effects [13,14]. A large body of literature has shown that SLs possess anticancer properties by including cell death, inhibiting proliferation, and limiting the spread of various malignancies in both in vitro and in vivo models. Key signaling pathways implicated in cancer progression, such as nuclear factor kappa B (NF-κB), signal transducer and activator of transcription 3 (STAT3), and Wnt/β-catenin, are modulated by these compounds [15]. One of the most abundant SLs in *L. rivularis* is leptocarpin (LTC) [12], a molecule that has displayed notable antitumoral activity on different cancer cell lines with low toxicity and high specificity [16].

In 2015, authors [16] demonstrated that LTC isolated from dried leaves reduces cell viability in various cancer cell lines, such as HT-29 (colon cancer), PC-3 (prostate cancer), DU-145, MCF7 (breast cancer), and MDA MB-231 (breast cancer). This inhibitory effect is dose-dependent, with a half-maximal inhibitory concentration (IC50) value ranging from 2.0 to 6.4 μM. Conversely, the impact on human dermal fibroblasts is considerably lower, with an IC50 of 18.7 μM.

The observed effects have been attributed to the induction of apoptosis, as evidenced by morphological alterations, significant fragmentation, and condensation of chromatin in cancer cells compared to controls. Additionally, LTC has been shown to induce depletion of mitochondrial membrane potential, leading to the release of cytochrome c and an increase in caspase-3 activity, promoting apoptotic processes. Cells treated with LTC exhibit a significant reduction in NF-κB activity at concentrations as low as 7 μM [16].

On the other hand, other authors [11] evaluated extracts from flowers using four solvents (n-hexane (Hex), dichloromethane (DCM), ethyl acetate (AcOEt), and ethanol (EtOH)), determining they were rich in sesquiterpenes, sesquiterpene lactones, caryophyllene oxide, dehydrocostus lactone, etc. The cytotoxicity of these extracts was evaluated in vitro against different cancer cell lines: HT-29 (colon cancer), PC-3 (prostate cancer), MCF-7 (breast cancer), and HEK-293 (nontumor cells) [11]. Their results indicated that, at nontoxic concentrations, all the extracts studied reduced the cellular viability of cancer cells in non-tumorigenic cells. Cancer cell line IC50 values were 3.0–8.8 µg/mL, except for ethanol extract, above 91 µg/mL. The lowest IC50 value (highest cytotoxicity) was in the HT-29 cell line. Flower extracts were much less cytotoxic against HEK-293, with IC50 values above 82.9 µg/mL [11].

Additionally, the flower extracts (except for those extracts dissolved in ethanol) were shown to act selectively against cancer cells. This effect was similar in magnitude to LTC but with a selectivity magnitude higher than that reported by Bosio et al [16]. The mechanism associated with these results was related to mitochondrial function, which showed an increase in mitochondrial membrane permeability in a concentration-dependent manner, similar to previous reports indicating that LTC induced caspase activation, causing cell apoptosis [11].

In 2023, authors [17] investigated the effect of *L. rivularis* flower extracts on proliferation, survival, and spread parameters of gastric cancer cells in vitro. Gastric cancer (AGS and MKN-45) and normal immortalized (GES-1) cell lines were treated with different concentrations of *L. rivularis* flower extracts (DCM, Hex, EtOAc, and EtOH). Extracts exerted a pronounced antiproliferative effect on both normal and cancer-derived gastric cells. The IC50 values were comparable across the different gastric cell lines tested, which was reflected in low selective index (SI) values. This outcome appears to be specific to gastric cells, as the same extracts showed SI values greater than 2 in other cell types, including breast (MCF7), prostate (PC-3), and colon (HT-29) [11]. Similar findings were obtained using purified LTC. Despite these results, concentrations near the IC50 were selected for each extract (DCM 5 µg/mL, EtOAc 5 µg/mL, Hex 10 µg/mL, and EtOH 15 µg/mL) to evaluate several parameters related to the malignant potential of gastric cancer cells and ensure reproducible biological effects. Under these conditions, the extracts demonstrated differential activity between cancerous gastric cells (AGS and MKN-45) and normal immortalized gastric cells (GES-1), at least in terms of cytotoxicity [17].

The extracts target multiple mechanisms essential for gastric cancer progression, including cell cycle regulation, mitochondrial function, clonogenic growth, and senescence induction. They also suppress migration and invasion in AGS and MKN-45 cells, key determinants of metastatic spread. In endothelial cells, EtOAc extract 1.0 µg/mL or leptocarpin at 2.5 µg/mL, their anti-angiogenic activity suggests a broader capacity to impair neovascularization. Collectively, these findings highlight the therapeutic promise of the extracts as multi-target agents against gastric cancer [17].

The documented anti-cancer properties of *L. rivularis* have fueled its growing use as an alternative medicinal source, generating a high demand for harvesting from its native ecological environment. Although there is no evidence of overexploitation, some efforts have been reported regarding vegetative propagation [18] and in vitro clonal micropropagation as a promising method for cultivation under controlled laboratory conditions [19]. A study [20] successfully established clonal micropropagation of *L. rivularis* and generated callus cultures from the internodal segment in vitro. The phytochemical analysis of micropropagated plants shared similar profiles with those collected from natural habitats, with LTC identified as the major component. However, LTC was absent in callus-derived extracts.

**Figure 2:**
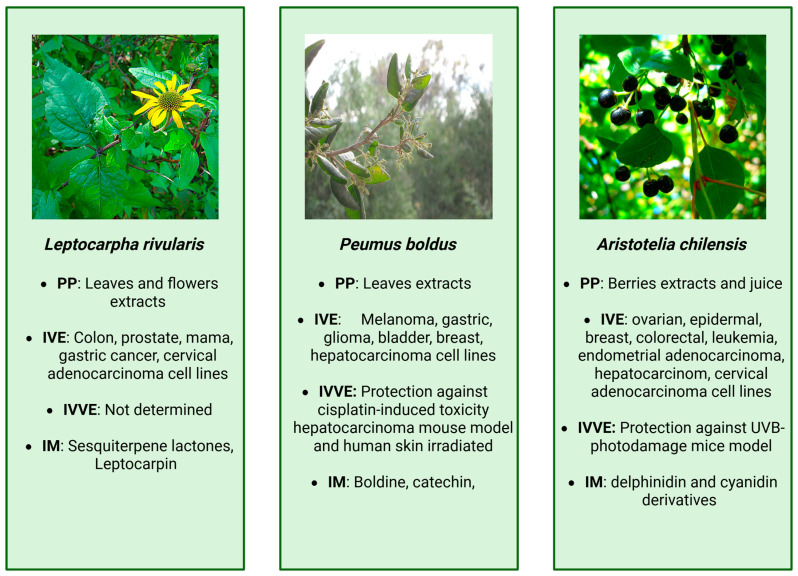
Summary of the main findings associated with *Leptocarpha rivularis*, *Peumus boldus*, and *Aristotelia chilensis*, images provided by Wikimedia [21,22,23]. PP: part of plant, IVE: in vitro evidence, IVVE: in vivo evidence, IM: identifies molecules.

The cytotoxicity of extracts obtained from in vitro-propagated *L. rivularis* was evaluated using both cancer and normal cell lines, including HeLa (cervical adenocarcinoma) and CCD841/CoN (normal colon epithelium) at 12 ppm. Cell viability and the expression of *IL-6* (interleukin-6) and *MMP2* (matrix metalloproteinase 2), genes associated with carcinogenic activity, were measured in the presence and absence of LTC and the extracts of micropropagated *L. rivularis* plants. The results showed that both LTC and the extracts exhibited comparable antiproliferative effects, with a more pronounced reduction in viability and gene expression in cancer cells compared to normal cells [20]. This previously unreported activity suggested that *L. rivularis* extracts and LTC may act through dual mechanisms, one that directly affects NF-κB activity and regulates the cell cycle, and another that affects the activity of metalloproteinases, thereby inhibiting metastasis progression [15]. The main findings from extracts derived from *L*. *rivularis* are summarized in Figure 2 and detailed in Table 1.

### 3.2. Pemus boldus

*Peumus boldus* Mol. (Monimiaceae), commonly known as “boldo,” is an endemic tree of central Chile, dominating the landscape of many parts of the Mediterranean climate zone [38]. Traditionally used in Mapuche medicine, boldo has been extensively studied for its alkaloid content and has been successfully used in homeopathy and herbal medicine. It has been employed to treat digestive disorders, as a laxative and diuretic, to support liver functions, and to stimulate the production of bile in the gallbladder [39]. Historically, boldo leaves have been used to alleviate ailments now recognized as involving cytoprotective and anti-inflammatory responses, as well as gastrointestinal disorders [40]. The use of boldo leaves and the species discussed in this review for homeopathic purposes is a contentious and scientifically debatable issue. Many controversies and misconceptions surround the safety and efficacy of homeopathy. However, this review aims to bring scientific understanding closer to the findings of oral/historical and homeopathic traditions.

Boldo leaves contain 0.4–0.5% of at least 17 alkaloids belonging to the large benzylisoquinoline-derived family, along with essential oils of complex composition, tannins, and flavonoids such as catechin, kaempferol, quercetin, and their glycosides (e.g., rutin) [24,41,42,43,44]. Catechin is the most abundant flavonoid, and together with the alkaloid boldine, is the main contributor to the antioxidant activity of boldo extracts [41,42]. Due to the high catechin content and its bioactivity, authors [42] have suggested that quality control of boldo leaves should include the combined analysis of catechin and the characteristic aporphine alkaloids [42].

Boldine, ((S)-2,9-dihydroxy-1,10-dimethoxy-aporphine), is the major alkaloid in boldo leaves, present at approximately 0.12% [45]. It has attracted attention due to its potent antioxidant properties, preventing both enzymatic and non-enzymatic damage to biological systems. In vitro studies have shown that boldine inhibits the free-radical-mediated initiation and propagation of oxidative damage in membranes, observed in liver homogenates, hepatic microsomes, and erythrocyte ghosts. It also protects against free-radical-dependent lysis of red blood cells and hepatocytes [46,47,48]. Beyond its antioxidant activity, boldine has been reported to exert anti-inflammatory, hepatoprotective, antitrypanosomal (against *Trypanosoma brucei brucei*), and cytotoxic effects (on HeLa cells in vitro) [49,50].

A study conducted in 2011 [24] investigated the protective effect of a methanolic extract of *P. boldus* leaves in deoxyribonucleic acid (DNA) damage produced by ultraviolet (UV) and nitric oxide (NO), and their impact on the growth of human melanoma M14 cells and normal human non-immortalized buccal fibroblasts. Using High-Performance Liquid Chromatography (HPLC), the extract was found to contain boldine, catechin, quercetin, and rutin. The methanolic extract inhibited human melanoma cell line (M14) cell growth at concentrations of 5–40 µg/mL, while showing no cytotoxicity toward normal fibroblasts. Pure compounds alone did not affect cancer cell viability even at concentrations comparable to those in *P*. *boldus* extract, except catechin at 25 and 50 µM, which inhibited M14 cell growth. These findings suggested a synergistic interaction between boldine and flavonoids [24].

*P. boldus* extract increased lactate dehydrogenase (LDH) release in M14 at higher extract concentrations (20 and 40 µg/mL) and induced apoptosis-mediated cell death, with caspase-3 activation observed at 5 and 10 µg/mL. In contrast, normal fibroblasts exhibited no cytotoxicity, as LDH release remained at control levels, at 20 and 40 µg/mL. But the levels of HSP70 (heat shock protein 70) protein decreased in M14 cells treated with methanolic extract at 5–20 µg/mL, with undetectable expression observed at 40 µg/mL. Since HSP70 is known to inhibit apoptosis and promote tumorigenicity, its reduction suggested a potential pro-apoptotic effect. In normal human fibroblasts, HSP70 expression remained unchanged across all tested concentrations (5–20 µg/mL). Collectively, these results indicated that methanolic extracts of boldo selectively inhibited the growth of melanoma cells while sparing normal cells [24].

Ethanolic boldus extracts (BE) of *Peumus boldus* have been reported to exert anti-hepatotoxic effects against cisplatin-induced damage in normal liver cells, both in vitro and in vivo, while preserving the cytotoxic activity of cisplatin against hepatocarcinoma cells [25]. For in vivo experiments, Swiss mice were used. To induce liver cancer, mice were treated with benzo [a]pyrene (50 mg/kg body weight (bw)) suspended in olive oil twice a week for one month and then kept on a normal diet for 2 more months during the onset and development of liver cancer. After the development of cancer, drug treatments (cisplatin and Boldo alone and combined) were conducted for 1 month. Cisplatin (10 mg/kg bw) was administered by intraperitoneal injection, and BE (40 mg/kg bw) orally once daily. The co-administration of BE with cisplatin increased the viability of normal cells without altering cancer cell survival. In vivo, boldo protected the liver from damage and normalized different antioxidant enzyme levels, which were determined by alanine aminotransferase, aspartate aminotransferase, lactate dehydrogenase, and glutathione assays using serum of mice. In vitro, 32–64 µg/mL of BE, it reduced reactive oxygen species (ROS) and re-polarized mitochondrial membrane potential. These protected effects were accompanied by reduced Bax and cytochrome c translocation, as well as caspase 3 downregulation. Furthermore, a drug-DNA interaction study revealed that BE reduced the DNA-binding capacity of cisplatin, thereby reducing DNA damage [25].

Other authors [26] showed that aqueous extracts of boldo had potent anti-urease activity and anti-adherent effects against *Helicobacter pylori*, which has been classified by the International Agency for Research on Cancer (IARC) as a type I carcinogen based on epidemiologic data [51]. The aqueous extracts from dried *P. boldus* leaves showed strong inhibition of *H. pylori* urease, an enzyme essential for bacterial survival in the gastric environment (IC50 = 144.4 µg/mL). A bioassay-guided fractionation identified fraction F5 as the most active against *H. pylori* urease with an IC50 = 15.9 µg gallic acid equivalents (GAE)/mL. HPLC analysis evidenced that F5 was composed mainly of catechin-derived proanthocyanidins. Both the aqueous extract and F5 reduced bacterial adhesion to AGS human gastric adenocarcinoma cells in a concentration-dependent manner, achieving up to 89.3% inhibition at 2.0 mg GAE/mL of boldo extract [26].

The effect of boldine, the major alkaloid of boldo, on glioma proliferation and cell death was evaluated [27]. Boldine decreased the cell numbers in U138-MG, U87-MG, and C6 glioma lines at concentrations of 80, 250, and 500 μM. Necrotic cell death was observed in the C6 lineage but not at lower concentrations in U138-MG and U87-MG lines, indicating that cell death might depend on both cell type and boldine dosage. Boldine treatment induced Gap 2 phase (G2) arrest in U138-MG cells and decreased the mitotic index, indicating a reduction in the proportion of cells undergoing mitosis, suggesting that boldine inhibits cellular proliferation in these gliomas [27]. Boldine treatment did not activate caspase-3 or caspase-9 in U138-MG, nor did it cause DNA fragmentation, indicators of apoptosis, suggesting that apoptosis was not induced through classical pathways. Comet assay confirmed the absence of DNA damage, contrasting with the extensive DNA damage caused by conventional chemotherapies such as temozolomide [27]. The observed non-genotoxic and selective cytotoxicity of boldine towards glioma cells, with negligible effects on normal brain cells, suggested its potential as a targeted therapeutic agent. Organotypic cultures exhibited toxicity only at high concentrations, reinforcing the promise of boldine as a safe candidate for in vivo studies [27].

Other authors [28,52] evaluated the effect of boldine on bladder cancer, one of the most prevalent genitourinary malignancies, and despite available chemotherapies, it remains associated with a high recurrence rate [53]. Boldine reduced cell viability and cell proliferation in the T24 human bladder carcinoma cell line, derived from a grade III human urinary bladder carcinoma at 200 to 500 μM. [28]. Mechanistically, boldine induced Gap 2/mitosis transition phase (G2/M) arrest and cell death, likely by disrupting proteins involved in G2-to-M transition. This effect was mediated primarily through the inactivation of Extracellular Signal-Regulated Kinase (ERK), halting cell cycle progression, and by Protein kinase B (or AKT) inactivation coupled with glycogen synthase kinase-3 beta (GSK-3β) activation, promoting apoptosis. Since ERK supports cell proliferation and AKT enhances cell survival, the dual targeting of boldine on these pathways represents a promising strategy to prevent cancer growth and promote cancer cell death [54,55,56].

Boldine markedly reduced the viability of invasive breast cancer cell lines (MDA-MB-231), inducing cytotoxicity and apoptosis through increased LDH, membrane permeability, and DNA fragmentation, showed at IC50 between 46.5 ± 3.1–70.8 ± 3.5 μg/mL. It also caused cell cycle arrest at the G2/M phase and disrupted mitochondrial membrane potential, leading to cytochrome c release and activation of caspase-9 and caspase-3/7. In MDA-MB-231 cells, boldine downregulated Bcl-2 and HSP70 while upregulating Bax, further enhancing apoptotic signaling. Additionally, boldine inhibited tumor necrosis factor alpha (TNF-α)-induced NF-κB activation, underscoring its potential as a multi-target agent against breast cancer progression and metastasis [29].

An acute toxicity study in rats demonstrated that boldine was well-tolerated at a dose of 100 mg/kg body weight. In this model, Rat LA7 mammary adenocarcinoma cells were injected into the mammary fat pads of female Sprague-Dawley rats to induce mammary cancer. Following intraperitoneal injection of boldine (50 or 100 mg/kg), tumor size was reduced in the breast cancer animal model, indicating its potential as a therapeutic agent for breast cancer [29]. Another study [30], boldine 90 mg/Kg/bw administered in drinking water in a rat model of hepatocellular carcinoma (HCC) induced by diethylnitrosamine, promoted caspase-dependent mitochondrial apoptosis. The study demonstrated that boldine modulated tumor and liver biomarkers and enhanced both enzymatic and non-enzymatic antioxidant activities. Histopathological examinations confirmed liver tissue improvement in HCC rats. Boldine activated the intrinsic apoptotic pathway by increasing Bcl-2, Bax, cytochrome c, and cleaved caspase-3 expression, thereby confirming its potent antiproliferative and pro-apoptotic properties in cancer cells [30].

Another study [57] showed that boldine exerted its antitumor activity through modulations of telomerase, an enzyme central to cell immortality and a key target in anticancer drug development. Telomerase, a ribonucleoprotein complex, extends the repetitive DNA sequences located at the ends of linear chromosomes, referred to as telomeres [58]. In normal somatic cells, telomerase activity is usually low or absent, resulting in gradual telomere shortening with each cell division [59]. However, in cancer cells, telomerase is frequently reactivated, enabling these cells to bypass the typical constraints on cellular replication and attain what is referred to as cellular immortality [60]. Telomerase function is primarily regulated at the transcriptional level of its catalytic subunit, TERT (Telomerase reverse transcriptase). Evidence has demonstrated the antiproliferative effects of boldine on multiple cancer cell lines at non-toxic concentrations, highlighting its potential as a telomerase inhibitor [57,61]. Boldine exhibits lower cytotoxicity in human fibroblasts compared to HEK293 and breast cancer (MCF-7 and MDA-MB-231) telomerase-positive cell lines. It inhibits telomerase activity in treated cells through down-regulation of hTERT, the catalytic subunit of the enzyme. Additionally, boldine influences hTERT splicing variants, promoting shorter, non-functional transcripts, potentially contributing to its inhibitory effect [57].

On the other hand, another study [31] showed that a novel synthetic derivative of boldine, N-benzylsecoboldine hydrochloride (BSB), exhibited telomerase inhibitory properties. The viability assays determined LD50 (50% of the maximal lethal dose) values for BSB of 16.25 µM for MCF7 cells and 21.88 µM for MDA-MB-231 cells. Consistent with these results, microscopic examination revealed a substantial, dose-dependent decrease in cell viability when exposed to BSB. The inhibitory effect of boldine on telomerase activity was dose-dependent, so that in the presence of 150 µM boldine, telomerase activity fell to around 10% of the control reaction, showing a calculated IC50 of 0.17 ± 0.1 and 68 ± 2.5 µM for BSB and boldine, respectively [31]. BSB has an IC50 roughly 400 times more potent than boldine. Although both compounds effectively bound the active site, molecular dynamics analysis suggested that BSB interacted with a secondary site through two hydrogen bonds, resulting in a stronger interaction than boldine. Theoretical evaluations also indicated that BSB could achieve submicromolar IC50 levels [31]. Due to its higher hydrophobicity and flexibility, BSB is a promising candidate for telomerase inhibition at non-toxic concentrations.

Poly (lactide-co-glycolide) (PLGA)-nanoparticles loaded with Boldine (named NBol) have been generated and tested in a hepatocarcinoma mouse model, Mus musculus, to examine if it could reduce unwanted Cisplatin-induced toxicity in normal tissue [32]. Cisplatin is a widely used anti-cancer drug with severe side effects precluding its sustained use. In comparison, the oral administration of NBol (10 mg/kg bw) has demonstrated superior drug delivery capacity and offered approximately 29% protection against cisplatin-induced toxicity. NBol efficiently entered cells, preventing cisplatin from intercalating with dsDNA, which resulted in reduced chromatin condensation, alterations in ROS levels, mitochondrial function, and antioxidant enzyme activities [32]. These effects contributed to decreased DNA damage and cell death. Expression analysis of apoptotic genes such as Top II, p53, Bax, Bcl-2, cytochrome c, and caspase-3 indicated that NBol enhanced cytoprotective effects in normal tissues [32]. However, more animal experiments might be necessary before recommending the use of this combined therapy in human trials for cancer patients who need and respond well to Cisplatin therapy.

Finally, boldine has been used as a sunscreen to block ultraviolet radiation (UVR), absorbing chemicals that attenuate the amount and nature of UVR reaching cells in the skin, preventing DNA damage from being generated in skin cells, which can lead to cancer [62]. An in vitro study evaluated the UV filtering capacity of natural substances, including boldine from *P. boldus*. Applied to volunteers and then irradiated with UV, the UV sun protection factor (SPF) was measured and compared to non-irradiated skin. Usnic acid exhibited the highest SPF (mean 4.1), followed by boldine (with a mean of 3.4). These values were comparable to the SPF of Nivea Sun Spray^®^ (mean 4.2) [62].

The main findings from extracts derived from *P. boldus* are summarized in Figure 2 and detailed in Table 1.

### 3.3. Aristotelia chilensis

*Aristotelia chilensis* (Mol.) Stuntz, commonly called maqui, is a dark fruit native to central and southern Chile and parts of Argentina [63,64]. Traditionally, its berries have been used in ethnomedicine to treat ailments such as sore throat, diarrhea, and dysentery [65]. In Chilean folk medicine, the leaves are valued for diverse properties, including astringent, febrifuge, anti-inflammatory, analgesic, anti-hemorrhagic, antioxidant, and cardioprotective effects [66,67,68,69].

Maqui fruit contains a diverse profile of polyphenols, including anthocyanins, flavonols, and ellagic acid. Delphinidin-based anthocyanins are the most abundant, while quercetin derivatives dominate among flavonols [70]. These compounds exhibit strong antioxidant activity and are linked to anticancer, antimicrobial, anti-inflammatory, and metabolic syndrome–related enzyme inhibition [71,72,73]. Among anthocyanins, flavonoids responsible for the colors of many vegetables, flowers, and fruits [74,75,76], the presence of delphinidin and cyanidin derivatives highlights the therapeutic potential of maqui as a source of bioactive molecules with broad medicinal relevance [77,78,79].

Anthocyanins from maqui, such as delphinidin and cyanidin-3-glucoside, have been shown to reduce proliferation in SKOV3 ovarian cancer cells and have a protective effect, preventing the apoptosis of UV-irradiated HaCaT cells (keratinocyte cells) [33,34], while cyanidin-3-sambubioside inhibits migration and invasion in MDA-MB-231 breast cancer cells [80]. Maqui extract also decreases inflammatory markers in colorectal adenocarcinoma and macrophage cell lines, supporting its anti-inflammatory potential [78,81,82,83]. Together, these findings suggest that anthocyanins contribute to the antiproliferative and anti-metastatic properties of maqui across diverse cancer types [77,78]. Beyond anthocyanins, the fruit contains additional polyphenols—including quercetin, myricetin, kaempferol, gallic acid, protocatechuic acid, ellagic acid, and p-coumaric acid—that further enhance its bioactivity [84,85]. The high phenolic yield obtained through hydroethanolic extraction underscores maqui’s value as a rich source of compounds with therapeutic relevance for cancer prevention and treatment [35].

The hydroethanolic extract decreased the viability of Ishikawa cells (endometrial adenocarcinoma cell line), with an EC50 of 472.3 µg/mL. Although it did not induce cell cycle arrest, the increase in apoptosis observed suggested that the decrease in cell viability might have been mediated through the activation of apoptotic pathways [35]. Since the extract did not affect cell cycle distribution but still decreased cell viability, it is suggested that the components of the extract induced cell death primarily through pro-apoptotic pathways without exerting cytostatic effects [35]. Previous studies have shown that anthocyanins from Hibiscus sabdariffa (mainly delphinidin 3-sambubioside and cyanidin 3-sambubioside) could decrease cell viability by arresting the cell cycle at the G2/M phase in HL-60 leukemia cells [80]. The hydroethanolic maqui extraction reduced by approximately 70% the invasion capacity of Ishikawa cells; however, it did not affect the migration capacity. Thus, besides reducing cell viability, the maqui extract reduced metastatic properties in endometrial cancer. Similarly, authors [86] reported that an extract of black rice (*Oryza sativa* L. *indica*) rich in cyanidin-3-glucoside reduced cell invasion in SKHep-1 and Huh-7 cells from human hepatocarcinoma, HeLa cells from human cervical carcinoma, and SCC-4 cells from human squamous cell carcinoma. Therefore, hydroethanolic maqui extract affected two important cancer hallmarks, such as apoptosis evasion and invasive capability in Ishikawa cells, allowing this traditional Chilean plant to become a potential source of therapeutic agents in endometrial cancer.

Authors [36] demonstrated that the methanol/water extracts rich in anthocyanins exhibited potent chemoprotective abilities in decreasing the growth of HT-29 and CaCo-2 colon cancer cells, with a 50% inhibition at concentrations higher than 50 μg/mL. Additionally, the methanolic extract increases the viability of macrophages RAW-264.7 at 20 μg/mL, also increasing the expression of inducible nitric oxide synthases (iNOS) and cyclooxygenase 2 (COX-2). It has been shown that a diluted juice from *Aristotelia chilensis* (AC) for 24 h decreased the protein and mRNA expression of COX-2, which is highly expressed in human colon cancer, and TNF-α-induced NF-κB activity and NFAT (nuclear factor of activated T-cells) activation in Caco-2 cells. Conversely, four hours after treatment, AC temporarily lowered cytoplasmic inhibitor kappa B alpha (IκBα) levels and increased phosphorylation of ERK1/2 and Akt, along with C-fos expression. This treatment also downregulated COX-2 expression; AC did not impact the viability of Caco-2 cells [81].

The potential of maqui extends beyond its antitumor effects, as its photoprotective properties have also been investigated [37]. They studied properties of the ethyl acetate fraction of maqui berry ethanol extract (MEE) in vitro and in vivo. MEE reversed UVB-induced DNA damage in HaCaT cells by enhancing antioxidant defenses such as superoxide dismutase, catalase, and glutathione, while reducing nitric oxide production. In BALB/c mice, MEE strengthened antioxidant capacity, lowered lipid peroxidation, and modulated cytokine levels by downregulating IL-6 and TNF-α and upregulating interleukin 4 (IL-4). Additionally, it inhibited ERK and p38 MAPK activation, supporting its effectiveness against UVB-induced photodamage [37].

Methanolic extracts of maqui (*Aristotelia chilensis*) present important inhibitory-like features against inflammation [87]. Investigated the effects of methanolic maqui extracts on RAW264.7 macrophages at a concentration of 100 μM (total polyphenolic content). The results showed that maqui extracts reduced nitric oxide production, inhibited the gene expression of inducible nitric oxide synthase and TNF-α, while concurrently enhancing the expression of interleukin 10 (IL-10). The main findings from *A. chilensis* extracts are summarized in Figure 2 and detailed in Table 1.

### 3.4. Drimys winteri

*Drimys winteri*, known as the Canelo tree, is revered in Chilean indigenous culture as the “sacred Mapuche tree” due to its healing, antibacterial, and disinfectant properties [88,89]. Traditional medicine uses leaf infusions to treat stomach ailments, bronchitis, allergies, asthma, and other inflammatory conditions, while it also supports therapies for respiratory issues, fever, neoplasms, and ulcers [90,91]. Essential oils from its leaves and bark exhibit notable pharmacological activities, including antifungal, immunomodulatory, anti-inflammatory, and anticancer effects [89,92].

*Drimys winteri* contains numerous monoterpenes, including α-pinene, β-pinene, linalool, and β-caryophyllene, as well as sesquiterpenes such as polygodial [89,93,94]. These bioactive compounds display antimicrobial, antioxidant, and antitumor properties, with essential oils from their leaves and bark showing activity against melanoma, breast, and prostate cancer cell lines [90,92,95]. In A375 melanoma cells, drimane sesquiterpenes from *Drimys winteri* bark inhibited cellular growth and induced apoptosis activity with the IC50 values of 305 ± 0.10 μg/mL (extract), 31.25 ± 0.045 μM (drimenol), 16.62 ± 0.027 μM (isonordrimenone), and 12.88 ± 0.023 μM (polygodial) [90]. The oils from the aerial parts of *D. winteri* also demonstrated selective antiproliferative effects in breast (MCF7) and renal (ACHN) cancer cells compared to normal cells, highlighting their therapeutic potential [92]. MCF-7 cells showed a decrease in proliferation in a concentration range from 16 to 64 μg/mL, and human epithelial renal cells (HK-2, 786-O, and ACHN) between 32 and 64 μg/mL.

In Drymis species, sesquiterpenes such as polygodial have also been identified, with extracts from *D*. *winteri* decreasing the viability of prostate (DU-145, PC-3) and breast (MCF-7) cancer cell lines [94]. Among these compounds, A semisynthetic derivative of polygodial (designated compound 8) stood out, showing the highest cytotoxicity with IC50 values = 70.6 ± 5.9 for DU-145, 65.4 ± 5.5 μM for PC-3, and 97.1 ± 7.2 μM for MCF-7 μM [94], reinforcing its therapeutic potential.

The monoterpenes α-pinene and β-pinene exhibit notable antitumor properties and show enhanced effectiveness when combined with chemotherapy drugs [96]. In vitro experiments using A-549 and H460 lung carcinoma cells revealed that while α-pinene and β-pinene alone did not suppress proliferation, their combination with paclitaxel significantly increased cytotoxic activity [97]. α-pinene and β-pinene have demonstrated tumor growth inhibition in several cancer models, including colon, prostate, liver, and lung cancers, and studies highlight their synergistic potential with paclitaxel [98,99,100].

**Figure 3:**
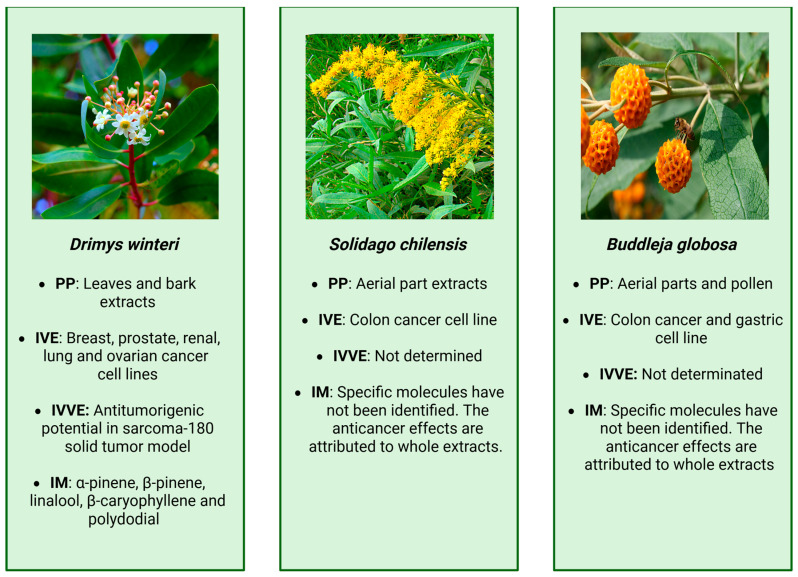
Summary of main findings related to *Drimys winteri*, *Solidago chilensis*, and *Buddleja globosa*, images provided by Wikimedia [102], Wikipedia [103], and Wikimedia [104], respectively. PP: part of plant, IVE: in vitro evidence, IVVE: in vivo evidence, IM: identifies molecules.

The monoterpene linalool has demonstrated notable antitumor activity, showing dose-dependent effects in vitro. Increase in cytotoxic effect of linalool in Sarcoma 180 cells between concentrations of 1.3–3.9 mM. In vivo, solid tumors produced by subcutaneous inoculation of Sarcoma-180 cells on the dorsal surface of the right hind leg of Swiss albino mice have been reduced in volume, weight, and cell count after linalool (150, 200, and 250 mg/kg) orally administered for 21 days [101]. Treatment with all the 3 doses of linalool resulted in a significant decrease in tumor volume, tumor weight, and tumor cell count most potent dose was found to be 200 mg/kg body weight. Although studies on *D*. *winteri* essential oil remain limited, its terpene-rich chemical profile suggests promising chemotherapeutic applications, warranting standardized in vitro and in vivo models for future biomedical research. The main findings from *D*. *winteri* extracts are summarized in Figure 3 and detailed in Table 2.

### 3.5. Solidago chilensis

*Solidago chilensis*, known as “vara dorada,” is a South American native plant traditionally used as an anti-inflammatory, diuretic, and remedy for gastrointestinal disorders [107]. Species of the Solidago genus contain diverse bioactive compounds, including terpenoids, saponins, phenolic acids, and high levels of flavonoids such as quercetin, kaempferol, and rutin [108,109]. Experimental studies have shown that Solidago extracts possess anti-inflammatory, antimicrobial, antineoplastic, analgesic, and antipyretic properties, highlighting their therapeutic potential [110,111,112,113,114,115,116,117,118,119].

The aqueous and hydroalcoholic extracts of the aerial parts of *S. chilensis* have anti-inflammatory [120,121,122,123] and hypoglycemic activities [124], hypolipidemic [125], analgesic effects in low back pain and tendinitis [126], and recently, the antiulcer activity [120], antidepressant [127]. Authors reported the anti-proliferative effect of *S. chilensis* infusions in the T84 colon cancer cell line [105]. Increasing the concentration of the extract decreased cell proliferation, showing an EC50 of 0.16 mg/mL. The aqueous extract studied exhibits high levels of antioxidant activity, and phenols and flavonoids, comparable to those of other reported species. The antiproliferative effect on cancer cells occurs at concentrations lower than those reported in similar works for other infusions of aromatic plants [105].

A few years later, the same group analyzed the aqueous extracts of *S. chilensis* [106]. They found that the values of QE (quercetin equivalent, phalvoneoid) for *S. chilensis* were considerably higher than those of Green Tea. Antiproliferative activity was assessed on T84 (tumoral) and HTR-8/SVneo (non-tumoral) cell lines, revealing that the percentage of viable cells declined as the concentration of the lyophilized *S. chilensis* extract increased in both cell types. The comparison of the EC50 values for both cell lines indicated that *S. chilensis* affected the proliferation of both cell lines in a similar way. Conversely, gallic acid was found in *S. chilensis* and could be responsible for the antiproliferative effects seen in these infusions. Gallic acid is the only compound identified with three neighboring hydroxyl groups in its structure, which grants it strong antiproliferative properties [128]. Despite these promising findings, more thorough studies are needed to elucidate further properties and mechanisms of action of *S. chilensis* as an anticancer agent. The main findings from S. *chilensis* extracts are summarized in Figure 3 and detailed in Table 2.

### 3.6. Buddleja globosa

*Buddleja globosa* Hoppe, known as matico, has long been valued in South American ethnomedicine for its wound-healing and anti-ulcer properties, with traditional uses extending to liver, gallbladder, and gastrointestinal disorders [129]. In folk medicine, preparations from its leaves and stems are applied as poultices or infusions to treat a wide range of conditions, including internal and external wounds, ulcers, liver and gallbladder pain, gastrointestinal disorders, dysentery, scabies, and syphilis [130,131,132]. Phytochemical studies reveal that *B*. *globosa* contains diverse bioactive compounds, including saponins, terpenoids, phenylethanoids, and flavonoids such as apigenin, quercetin, and hydroxyluteolin [129,133].

A study analyzed aqueous extracts of aerial parts of *B. globosa* because these plants are traditionally consumed as infusions and found antiproliferative and antioxidant properties [106]. Antiproliferative activity of the infusions was evaluated by exposing T84 (colon adenocarcinoma cell line) and HTR-8/SVneo (non-tumoral) to different concentrations of lyophilized extracts up to a maximum concentration of 5 mg of lyophilizate per mL of culture medium. These concentrations follow the quantities that can be obtained at the intestinal level, after dietary ingestion of 1–2 cups of herbal infusions [134]. In tumoral and non-tumoral cells, *B. globosa* extracts have a significant antiproliferative activity. The comparison of the EC50 values between the two cell lines revealed that the extracts exhibited different antiproliferative specificity, with values of 1.37 ± 0.17 mg/mL for T48 cells and 0.29 ± 0.09 mg/mL for HTR8-SVneo. *B. globosa* has a higher antiproliferative effect on HTR-8/SVneo cells than T84. This study showed that *B. globosa*, in comparison with Green Tea, presented higher values for the content of antioxidants (Vitamin C equivalent antioxidant capacity) and GAE phenols [106].

Recently, the pollen of *B. globosa* flowers has also become a subject of study, besides the leaves, which have been a source of anticancer research. Honeybee pollen (HBP) loads are a mix of flower pollen from different plant species, adhered to by nectar and enzymes secreted by the salivary glands of honeybees. Its chemical composition is highly variable, conforming to the floral and geographical origin of the pollen grains. The beneficial effects and functional properties of HBP are well-known and have been mainly attributed to their high content of antioxidant polyphenols. A study has shown that *B. globosa* is one species that provides pollen to the HBP [135]. HBPs from Los Lagos Region (X Region, southern Chile) are rich sources of phenolic antioxidants that exhibit a protective effect in vitro against DNA damage induced by peroxyl radicals, showing promising results (up to 91.2% protection). The polyphenol content and antioxidant capacity in HBPs achieved complete bio-accessibility, including after the intestinal digestion process. Therefore, the potential of HBPs in the prevention of gastric and/or intestinal cancer warrants further investigation. The main findings from *B. globosa* extracts and HPB are summarized in Figure 3 and detailed in Table 2.

### 3.7. Senecio graveolens

*Senecio graveolens* (Chachacoma), a high-altitude Andean herb, is traditionally used for wound healing, anti-inflammatory purposes, and to alleviate altitude sickness [136]. Its ethanolic extract and major compound 4-hydroxy-3-(3-methyl-2-butenyl) acetophenone showed cytotoxic activity against breast cancer cell lines (ZR-75-1, MCF-7, MDA-MB-231) but not in non-tumorigenic MCF-10F cells, indicating selective antitumor potential [137].

**Figure 4:**
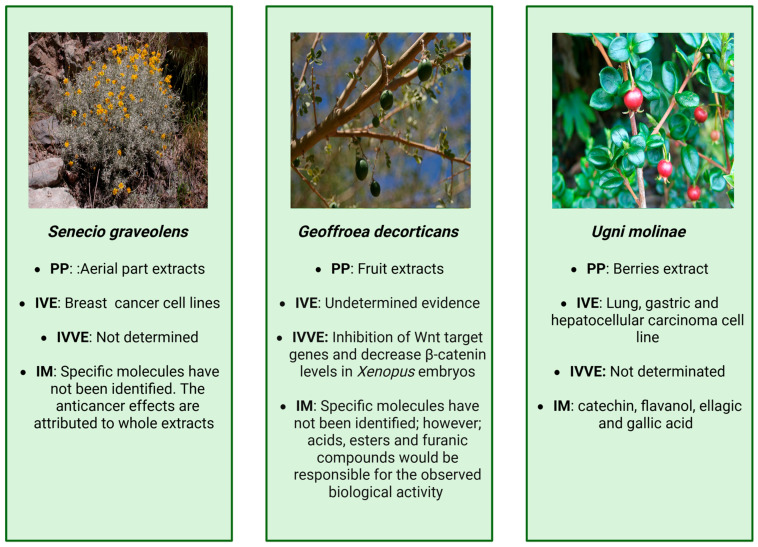
Summary of main findings associated with *Senecio graveolens*, *Geoffroea decorticans*, and *Ugni molinae*, images provided by Wikimedia [138,139,140]. PP: part of plant, IVE: in vitro evidence, IVVE: in vivo evidence, IM: identified molecules.

The phytochemical extract induced cytotoxicity in cancer cells but not in non-tumorigenic cells. The dose of 200 μg/mL reduced the cell viability by over 90% in MCF-7 and ZR-75-1 cells (tumor cell lines). This effect was enhanced under hypoxic conditions; however, 4-hydroxy-3-(3-methyl-2-butenyl) acetophenone did not, by itself, show an effective anticarcinogenic activity compared to the whole extract [137]. The cytotoxic effect of the *S. graveolens* extract depended on the basal superoxide dismutase (MnSOD) protein expression, an enzyme that removes superoxide radicals (O_2_•^−^) and produces hydrogen peroxide (H_2_O_2_) [137]. Therefore, cytotoxicity was greater when MnSOD levels were low; conversely, elevated MnSOD levels conferred resistance, enabling cells to survive more effectively to exposure to *S. graveolens*. Additionally, the results showed that exposure to *S. graveolens* extract led to overexpression of activated caspase-8 in MCF-7 cells and a decrease in MDA-MB-231 cells. Cleaved caspase-3 was detected in ZR-75-1 cells, indicating apoptosis initiation, whereas MDA-MB-231 cells showed increased pro-caspase-3 without activation [137]. On the other hand, MAP LC3β was upregulated in MCF-7 cells but not detected in ZR-75-1 cells, and both MAP LC3α and MAP LC3β were downregulated in MCF-10F and MDA-MB-231 cells. MAP LC3α and β proteins indicated autophagosome formation. These results suggest that the crude extract seems to trigger cell death by a variety of processes, including autophagy, apoptosis, and necrosis, especially in MCF-7 cells, although further research is needed to determine their relative roles. Despite the findings regarding the cytotoxic activity of *S. graveolens*, further studies are needed to understand the effects of these phytochemical compounds on cancer [137]. The main findings from *S. graveolens* extracts are summarized in Figure 4 and detailed in Table 3.

### 3.8. Geoffroea decorticans

The *Geoffroea decorticans* (commonly named chañar) is a vegetal species widely distributed in the center and north of Argentina, north of Chile, south of Peru, and Bolivia. Its fruits (drupes) and products are commonly used for both culinary and traditional medicinal purposes by rural communities [145]. The most popular product made from chañar fruits is a sweet jelly-like syrup called arrope, used both as a sweetener and as a cough syrup in traditional medicine [146]. The identification of polyphenolic compounds from *G. decorticans* exhibiting antioxidants, anti-inflammatory, and anti-nociceptive properties has led to the elucidation of certain mechanisms of action, including potential anti-carcinogenic effects [146,147,148].

There are reports demonstrating that the anti-tumor effects of natural compounds are related to their ability to modulate the Wnt/β-catenin signaling pathway [149,150,151]. A study conducted in 2021 chemically characterized three *G. decorticans* extracts and determined their capacity to modulate the Wnt/β-catenin pathway [141]. Aberrant activation of the Wnt/β-catenin cell signaling pathway can cause various pathologies, including cancer. Recently, *Xenopus laevis* embryos have become a very interesting strategy to screen natural molecules and their effects on the Wnt/β-catenin signaling pathway [150,152,153].

Another study [141] used *G. decorticans* extracts of *Xenopus* embryos to study regulators of signaling pathways involved not only in embryonic development but also in carcinogenic processes. This work showed that *G. decorticans* extracts exhibited predominant acids, esters, and furanic compounds that affect axis formation in *Xenopus* embryos, producing ventralized phenotypes. Additionally, *G. decorticans* extracts suppressed the expression of Wnt target genes and decreased β-catenin levels in *Xenopus* embryos. These results indicated that *G. decorticans* could be a source of β-catenin inhibitory compounds with potential applications in cancer treatment. These findings open the possibility of further research into the potential of *G. decorticans* and their effects against cancer. The main findings from *G. decorticans* extracts are summarized in Figure 4 and detailed in Table 3.

### 3.9. Ugni molinae

The *Ugni molinae* (Murta), a native Myrtaceae from south-central Chile, produces small aromatic berries rich in diverse phenolic compounds, including flavonols, phenolic acids, and anthocyanins [154,155,156,157,158]. While research has largely focused on their antioxidant properties and phytochemical composition, extracts have also demonstrated in vitro inhibition of cancer cell proliferation, with compounds such as delphinidin-3-rutinoside, chlorogenic acid, and epicatechin contributing to anticancer and anti-stress effects [159,160,161,162]. Recent studies further show that different drying methods—freeze, vacuum, infrared, convective, and sun drying—can influence the retention of bioactive compounds and thereby affect antioxidant, anti-inflammatory, and antitumor activities [163].

Fresh *U*. *molinae* berries contain catechin as the predominant phenol, while gallic acid and 3-hydroxytyrosol are present in lower amounts; in dehydrated samples, pyrogallol becomes the most abundant, especially under convective drying [142,163]. Cytotoxic assays showed that extracts at 0.25 mg/mL (or 250 µg/mL) in phosphate-buffered saline reduced cell viability in human lung carcinoma (NCI-H1975) and mouse neuronal immortalized (HT-22) cells. Fresh extracts lowered viability by about 13% in NCI-H1975, and freeze-dried samples showed the strongest effect in HT-22 [142,143]. Among drying methods, vacuum and infrared drying best preserved the antitumor activity, likely due to phenolic compounds with oxidative capacity, suggesting that *U*. *molinae* extracts selectively impair cancer cell survival [143,163].

Chemical analyses of *U*. *molinae* extracts revealed a rich polyphenol profile, with catechin as the predominant compound [164]. Catechin has demonstrated anticancer activity across multiple systems, including inhibition of proliferation of immortalized murine hepatic cell line GRX through anti-inflammatory and cell cycle–related mechanisms, while other phenols such as ellagic and gallic acids showed cytotoxicity against HepG2, HCT 116, and colon tumor cells [165,166,167]. Further studies reported that antioxidant-rich berry and leaf aqueous extracts exert synergistic effects against colon and gastric adenocarcinoma cell lines. Metabolites such as Tannins and flavonoids reduce cell viability in AGS human gastric adenocarcinoma to 62.5 µg/mL, underscoring their therapeutic potential [144]. The main findings from *U. molinae* extracts are summarized in Figure 4 and detailed in Table 3.

### 3.10. Austrocedrus chilensis

*Austrocedrus chilensis* (D. Don) is a species belonging to the Cupressaceae and is endemic to the southern portion of South America, naturally being found only in Chile and Argentina. The wood of this plant is well known for its durability and ability to resist biological degradation [168,169]. Evidence suggests this wood quality is due to a specific class of secondary metabolites [170].

**Figure 5:**
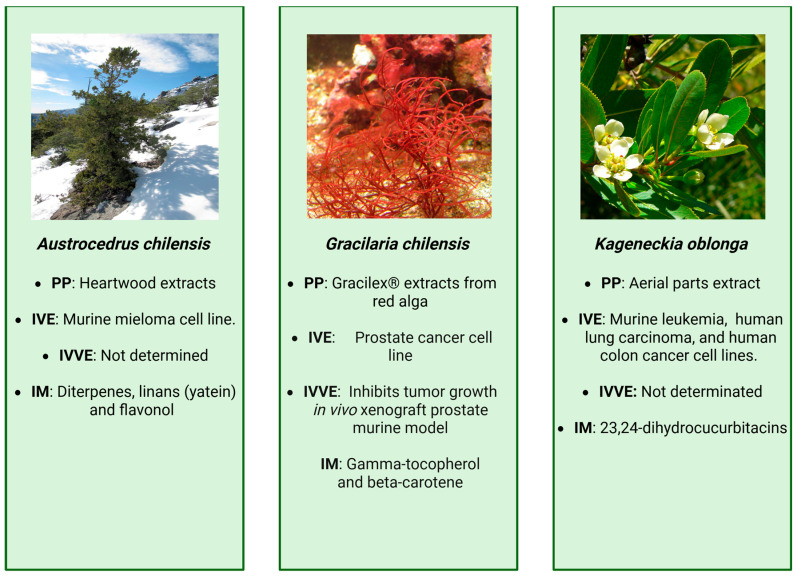
Summary of main findings related to *Austrocedrus chilensis*, *Gracilaria chilensis*, and *Kageneckia oblonga*, images provided by Wikimedia [174,175] and Wikipedia [176], respectively. PP: part of plant, IVE: in vitro evidence, IVVE: in vivo evidence, IM: identified molecules.

The secondary metabolites found in extracts of *A. chilensis*, such as diterpenes and lignans, have been isolated and chemically characterized, revealing these compounds as major constituents [170,171]. It is well recognized that lignans and diterpenes are involved in plant defense mechanisms and possess biological activity, exhibiting properties such as antibiotic, antifungal, antiviral, immune-modulating, antiasthmatic, antioxidant, antineoplastic, gastroprotective, and cytotoxic effects [172]. In 2013, a study [173] evaluated the antiproliferative activity of a diterpene (ferruginol) and a flavonol (isorhamnetin) isolated from *A. chilensis* and elucidated their cytological effects on P3X murine myeloma cells. The results showed that Yatein, a lignan isolated from *A. chilensis*, potentially inhibited P3X murine myeloma cell proliferation, resulting in approximately 75% cell death in response to a 25 µg/mL treatment. The P3X cells lost membrane integrity at the nuclear and cytoplasmic levels, including organelles, in response to Yatein treatment (12.5 µg/mL). Additionally, changes in the cytoplasmic organization and distribution of microtubules were observed. The other compounds tested had low activity [173]. These results are relevant because Yatein is a lignan precursor of podophyllotoxin, a key agent in anticancer drugs. Due to its structural similarities to podophyllotoxin, Yatein may act on similar cytoplasmic target (s), including the microtubular apparatus. Consequently, Yatein emerges as a compound of considerable pharmacological interest, warranting further evaluation in human cell lines. The main findings from *A. chilensis* extracts are summarized in Figure 5 and detailed in Table 4.

### 3.11. Gracilaria chilensis

*Gracilaria chilensis* (“pelillo”), a Chilean red macroalga, has been used since pre-Hispanic times as both food and medicine, and its nutritional value supports applications in cosmetics, the food industry, and biomedicine through agar extraction [179,180]. An oily extract known as Gracilex^®^ has been characterized as a source of natural peroxisome proliferator-activated receptor gamma (PPARγ) ligands and antioxidants, with potential to mitigate metabolic disorders due to its strong antioxidant activity [179]. Experimental studies demonstrated that Gracilex^®^ exerts time- and dose-dependent cytotoxic effects on prostate cancer cell lines (LNCaP and PC-3), reducing viability by more than 50%, inducing apoptosis via caspase-3 activation, and decreasing proliferation markers such as Ki-67 with an IC50 = 60 µg/mL [177]. In a xenograft model, male NOD-scid IL2Rgammanull mice were subcutaneously injected with PC-3 cells in their flanks. Gracilex^®^ treatment began five days after tumor cell injection and was administered via oral gavage three times per week for five weeks at a dose of 300 mg/kg body weight. On day 32 post-implantation, tumors were surgically harvested and processed for histological analysis. Gracilex^®^ inhibited migration, invasion, and tumor growth in xenograft models, reinforcing its therapeutic promise against prostate cancer [177].

The anticancer activity of Gracilex^®^ is attributed to its chemical composition, particularly its high gamma-tocopherol content, a potent vitamin E form with chemosensitizing and antiproliferative effects in prostate cancer models [179,181,182,183,184,185]. It also contains beta-carotene, a precursor of retinoic acid, which can regulate gene expression in prostate cancer cells [186]. Collectively, these bioactive compounds account for the broad antitumoral effects of Gracilex^®^ observed in vitro and in vivo, underscoring its therapeutic potential and the need for further research into algae-derived agents for cancer treatment. The main findings from *G. chilensis* extracts are summarized in Figure 5 and detailed in Table 4.

### 3.12. Kageneckia oblonga

*Kageneckia oblonga* is a native species that grows in central Chile. In folklore medicine, stems and leaves have been widely used in traditional medicine, mainly in infusion, as a potion to treat hepatic and kidney disorders and many other ailments [187]. From this plant, common triterpenoids, prunasin, and 23,24-dihydrocucurbitacin F, have been isolated and identified [178]. The first cytotoxic studies carried out with different extracts showed non-cytotoxic effects when tested on several neoplastic cell lines and the global ethanolic extract had nonspecific cytotoxicity against 3 tumor cell lines [187]; however, a study [178] reported that the global methanolic extract of the aerial parts of this plant (IC50 of 2.5 μg/mL) contained 23,24-dihydrocucurbitacins, bioactive compounds that exhibited cytotoxicity against P-388 murine leukemia, A-549 human lung carcinoma, and HT-29 cell lines.

The 23,24-dihydrocucurbitacin is a tetracyclic triterpenoid belonging to the cucurbitacin family. Various studies have demonstrated that cucurbitacin analogues have a broad range of biological effects, including anti-inflammatory, hepatoprotective, anti-cancer, and antioxidant activities [188]. These compounds have the potential to be used as possible bioactive agents to inhibit cancer progression, and these compounds contain structural improvements for potential chemotherapeutics. However, more specific research must determine its potential as an anticancer agent. The main findings from *K. oblonga* extracts are summarized in Figure 5 and detailed in Table 4.

## 4. Current Limitation and Future Direction

This work provides an update on the natural compounds derived from Chilean species and their cytotoxic potential against cancer. These species have primarily been utilized based on ancestral traditions and the knowledge of native peoples passed down through generations. For a long time, the mechanisms by which these species exert their cytotoxic effects were not well understood, and despite the efforts of various research teams globally, much remains to be discovered. The lack of standardized extracts or purified compounds and their storage stability, added to the variability in plant composition caused by geographic origin and seasonal factors, are some of the limiting aspects of this work. On the other hand, the extracts of most of the species mentioned have not been completely characterized, which presents pharmacotherapeutic limitations. The extracts or isolated compounds discussed may potentially affect other healthy cells or exert systemic effects in the bloodstream; therefore, further detailed studies are needed. The use of isolated molecules or extracts poses significant challenges due to the limited in vivo data and the absence of human clinical studies. There are also translational hurdles between preclinical research and clinical application, including regulatory issues related to the differentiation between nutraceutical and pharmaceutical development. While much work remains before these findings can be translated into therapeutic applications, the empirical evidence supported by years of ancestral tradition offers promising prospects for researchers.

## 5. Conclusions

The rich biodiversity in Chile, characterized by its varied climates and landscapes, presents a wide array of natural products that may possess anticancer cytotoxic properties. Various parts of these species—including leaves, flowers, fruits, and heartwood—have been utilized, and different extraction methods have been explored. Among the twelve Chilean species examined, *Peumus boldus*, *Aristotelia chilensis*, *Drimys winteri*, and *Gracilaria chilensis* are the most studied in vivo. While some effects have been observed, the precise cellular mechanisms behind these antitumor properties have not been completely elucidated. Conversely, studies involving extracts and isolated compounds from species such as *Leptocarpha rivularis*, *Solidago chilensis*, *Buddleja globosa*, *Senecio graveolens*, *Geoffroea decorticans*, *Ugni molinae*, *Austrocedrus chilensis*, and *Kageneckia oblonga* are still in the early stages.

Although knowledge about the mechanisms of these species on tumor cells is just beginning, there is a great incentive for researchers, which is the empirical evidence supported by years of ancestral tradition using these products. Extracts and isolated compounds from these plants have demonstrated abilities to induce cell death, decrease cellular viability, diminish clonogenic growth, provoke a senescent phenotype, and even exert anti-angiogenic effects. These species show low toxicity and high specificity, working through mechanisms such as cell cycle arrest, apoptosis induction, chromatin fragmentation, and mitochondrial disruption.

The findings presented here create a basis for future investigations, enabling an ongoing characterization of the molecular composition of these species, their effects on various cancer types, and potential clinical applications. The rich biodiversity in Chile continues to offer a plethora of products from distinct species aimed at creating therapies for cancer treatment and prevention.

However, there are translational challenges when this type of work is considered, for instance, the boldine and maqui berry extracts that are already marketed as nutraceuticals; the clinical translation requires GMP-standard isolation, pharmacokinetics, and toxicity profiling. Regulatory hurdles include defining whether these compounds are dietary supplements or investigational drugs. For future directions, it is recommended to prioritize lead compounds such as leptocarpin, boldine, anthocyanins, drimenol, and γ-tocopherol. Preclinical signals exist in cancer types/models, such as colon, breast, prostate, melanoma, and glioma. Furthermore, synergistic approaches as boldine with cisplatin that induce hepatoprotection and maintain cytotoxicity, can be considered. In the future, these types of studies can include several kinds of experimental models, such as xenografts, organoids, and co-culture systems, to validate pathway-specific effects. If the current descriptions have mechanistic gaps linking each plant/compound to a defined pathway, it would be necessary to create a coherent mechanistic framework, such as telomerase, oxidative stress, and other processes.

## Figures and Tables

**Table 1:** Main findings about *Leptocarpha rivularis*, *Peumus boldus*, and *Aristotelia chilensis* and their cytotoxic potential against cancer. Abbreviations. N.D: Not determined.

Plant Species (Common Name) Reference	Part Used	Extract Isolated Molecule	In Vitro/In Vivo Effect	Phytochemical Bioactive	Determined Concentrations
*Leptocarpha* *rivularis* (Palo negro) Montenegro I et al. (2020) [11], Bosio C et al. (2015) [16], (Palo negro Continued) Carrasco N et al. (2023) [17], Rubio J et al. (2022) [20].	Leaves	Isolated molecule	Reduces cell viability in HT-29 (colon cancer), PC-3 (prostate cancer), DU-145, MCF7 (breast cancer), and MDA MB-231 (breast cancer)	Leptocarpin	IC50 = 2.0–6.4 μM
	Flowers	n-hexane (Hex), dichloromethane (DCM), ethyl acetate (AcOEt)	Highest cytotoxicity in HT-29 (colon cancer), PC-3 (prostate cancer), MCF-7 (breast cancer)	N.D.	IC50 = 3.0–8.8 µg/mL
	Flowers	n-hexane (Hex), dichloromethane (DCM), ethyl acetate (AcOEt), and ethanol (EtOH)	Antiproliferative effect on cancerous gastric cells (AGS and MKN-45)	N.D.	DCM 5 µg/mL, EtOAc 5 μg/mL, Hex 10 μg/mL, and EtOH 15 μg/mL
			Anti-angiogenic activity in EA.hy926 endothelial cells	N.D.	EtOAc extract 1.0 µg/mL or leptocarpin at 2.5 µg/mL
	Whole plant and callus	Extracts obtained from in vitro-propagated *L. rivularis* in ethyl acetate.	Antiproliferative effects in HeLa (cervical adenocarcinoma) and CCD841/CoN (normal colon epithelium)	N.D.	Effect shown at 12 ppm (or 12 µg/mL)
*Peumus boldus* (Boldo) Russo A et al. (2011) [24], Mondal J et al. (2014) [25], Pastene E et al. (2014) [26], Gerhardt D et al. (2009) [27], Gerhardt D et al. (2014) [28], Paydar M et al. (2014) [29], Subramaniam N et al. (2019) [30], Kazemi Noureini S et al. (2018) [31], Mondal J et al. (2020) [32]. (Boldo Continued)	Leaves	Isolated molecule	Antiproliferative effects in U138-MG, U87-MG, and C6 glioma cell lines	Boldine	Effect shown at 80, 250, and 500 μM.
		Isolated molecule	Reduced cell viability in T24 human bladder carcinoma and MDA-MB-231 breast cancer cell lines	Boldine	For T24 between 200–500 μM For MDA-MB-231 IC50 between 46.5 ± 3.1–70.8 ± 3.5 μg/mL
		Isolated molecule	Reduced tumor size in the breast cancer and hepatocarcinoma animal model	Boldine	Intraperitoneal injection of 50 or 100 mg/kg/bw for breast cancer and 90 mg/kg/bw administered in drinking water for hepatocarcinoma
	Leaves	Isolated molecule	Reduce unwanted Cisplatin-induced toxicity in normal tissue in the hepatocarcinoma mouse model.	(PLGA)-nanoparticles loaded with Boldine.	Oral administration of NBol at 10 mg/kg bw
		Isolated molecule	Decreased viability in breast cancer cell lines (MCF7 and MDA-MB-231) and telomerase inhibitory properties	N-benzylsecoboldine hydrochloride (BSB)	LD50 for BSB of 16.25 µM in MCF7 cells and 21.88 µM in MDA-MB-231 cells
	Leaves	Methanolic extract	Antiproliferative effects in human melanoma cell line (M14)	Boldine, catechin, quercetin, and rutin	Effect shown between 5–40 µg/mL
		Isolated molecule	Antiproliferative effects in human melanoma cell line (M14)	Catechin	Effect shown at 25 and 50 µM
		Ethanolic extract	Anti-hepatotoxic effects against cisplatin-induced damage in normal liver cells, preserving the cytotoxic activity of cisplatin against hepatocarcinoma cells	N.D.	In vitro, effect between 32–64 µg/mL. In vivo, Swiss mice with induced liver cancer were treated with intraperitoneal Cisplatin and BE (40 mg/kg bw) orally once daily
		Aqueous extracts	Protective agent against the adherent and anti-urease activity of *Helicobacter pylori*, a type I carcinogen	Catechin-derived procyanidins	IC50 = 144.4 µg/mL
*Aristotelia* *chilensis* (Maqui) He Y et al. (2017) [33], Lim W et al. (2017) [34], (Maqui Continued) Mena J et al. (2021) [35], Céspedes-Acuña C et al. (2018) [36], Chen L et al. (2020) [37]	Berry (fruit)	Isolated molecule	Protective effect, preventing the apoptosis of UV-irradiated HaCaT cells (keratinocyte cells)	Cyanidin-3-O-glucoside	80–200 μM
		Isolated molecule	Antiproliferative effect in SKOV3 ovarian cancer cells	Delphinidin	0.1–100 µM
		Hydroethanolic extracts	Decreased the viability and invasion capacity of Ishikawa cells (endometrial adenocarcinoma cell line)	N.D.	EC50 = 472.3 µg/mL.
		Methanol/water extracts	Antiproliferative effects of HT-29 and CaCo-2 colon cancer cells,	N.D.	EC50 = 50 μg/mL.
		Ethyl acetate fraction of maqui berry ethanol extract	-Reversed UVB-induced DNA damage in HaCaT cells by enhancing the antioxidant defense system -Improves antioxidant capacity, lowers lipid peroxidation, and reduces inflammation in BALB/c mice exposed to UVB radiation	N.D.	Not accessible

**Table 2:** Main findings about *Drimys winteri*, *Solidago chilensis*, and *Buddleja globosa* and their cytotoxic potential against cancer. Abbreviations. N.D: Not determined.

Plant Species (Common Name) Reference	Part Used	Extract Isolated Molecule	In Vitro/In Vivo Effect	Phytochemical Bioactive	Determined Concentrations
*Drimys winteri* (Canelo) Russo A et al. (2019) [90], Bruna F et al. (2022) [92], Montenegro I et al. (2014) [94], Jana S et al. (2014) [101]. (Canelo Continued)	Bark	Essential oils	Inhibits cellular growth and induces apoptosis activity in A375 melanoma cells	Drimenol, isonordrimenone, and polygodial	IC50 values of 305 ± 0.10 μg/mL (extract), 31.25 ± 0.045 μM (drimenol), 16.62 ± 0.027 μM (isonordrimenone), and 12.88 ± 0.023 μM (polygodial)
	Aerial parts	Essential oils	Selective antiproliferative effects in breast (MCF7) and renal (ACHN) cancer cells compared to normal cells.	N.D.	For MCF-7: 16–64 μg/mL and ACHN between 32–64 μg/mL.
	Aerial parts	Isolated molecule	Decreasing the viability of prostate (DU-145, PC-3) and breast (MCF-7) cancer cell lines	Semisynthetic derivative of polygodial (designated compound 8)	IC50 values = 70.6 ± 5.9 for DU-145, 65.4 ± 5.5 μM for PC-3 and 97.1 ± 7.2 μM for MCF-7 μM
	Synthetic	Isolated molecule	-Increase in cytotoxic effect of linalool in Sarcoma 180 cells -Reduced volume, weight, and cell count in the Sarcoma 180 mice model	Linalool	Effect shown between 1.3–3.9 mM for cells 150, 200, and 250 mg/kg/wb orally administered
*Solidago chilensis* (Vara dorada) Gastaldi B et al. (2016) [105]	Aerial parts	Aqueous extracts	Anti-proliferative effect of *S. chilensis* infusions in the T84 colon cancer cell line	N.D.	EC50 = 0.16 mg/mL or 160 μg/mL
*Buddleja globosa* (Matico) Gastaldi B et al. (2018) [106]	Aerial parts	Aqueous extracts	Antiproliferative activity of the infusions was evaluated by exposing T84 (lung tumoral)	N.D.	EC50 = 1.37 ± 0.17 mg/mL for T48 cells

**Table 3:** Main findings about *Senecio graveolens*, *Geoffroea decorticans*, and *Ugni molinae* and their cytotoxic potential against cancer. Abbreviations. N.D: Not determined.

Plant Species (Common Name) Reference	Part Used	Extract Isolated Molecule	In Vitro/In Vivo Effect	Phytochemical Bioactive	Determined Concentrations
*Senecio graveolens* (Chachacoma) Echiburu-Chau C et al. (2014) [137]	Flowers, leaves, stems	Ethanolicextract	Cytotoxic activity against breast cancer cell lines (ZR-75-1, MCF-7, MDA-MB-231) but not in non-tumorigenic MCF-10F cells	4-hydroxy-3-(3-methyl-2-butenyl) acetophenone is the main compound, but it does not show a cytotoxic effect comparable to the whole extract.	200 μg/mL
*Geoffroea* *decorticans* (Chañar) Somaini G et al. (2021) [141]	Fruits	Aqueous extract (GdAE)subextracts of lyophilized GdAE, named *Geoffroea decorticans* chloroformic extract (GdChE) and *Geoffroea decorticans* ethyl acetate extract (GdAcE)	Suppression of Wnt target genes and decrease β-catenin levels in *Xenopus embryos* (study model for this pathway in cancer)	2-furancarboxaldehyde-5-hydroxymethyl, Heptyl hexanoic acid3-hydroxycholan-12-one, 2-furancarboxylic acid, Hexanoic acid-1-cyclobutyl ester, and 5-acetoxymethyl-2-furaldehyde were identified, but it was not proven whether they are effective on their own.	GdAE 30 mg/mL, GdChE 1 mg/mL, and GdAcE 0.5 mg/mL.
*Ugni molinae* (Murta) Grzesik M et al. (2018) [142], Caprioli G et al. (2016) [143], Avello M et al. (2020) [144].	Fruits	Berries were dried by freeze drying (FD), vacuum drying (VD), infrared drying (IRD), convective drying (CD), and sun drying (SD), and suspended in phosphate-buffered saline	Decreased viability in human lung carcinoma (NCI-H1975) and mouse neuronal immortalized (HT-22)	Catechin, as the predominant compound identify	250 µg/mL
	Leaves	Aqueous extracts (1%), in water at 80 °C	Reducing cell viability in AGS human gastric adenocarcinoma	Tannins and flavonoids	Effects shown to 62.5 µg/mL

**Table 4:** Main findings about *Austrocedrus chilensis*, *Gracilaria chilensis*, and *Kageneckia oblonga* and their cytotoxic potential against cancer. Abbreviations. N.D: Not determined.

Plant Species (Common Name) Reference	Part Used	Extract Isolated Molecule	In Vitro/In Vivo Effect	Phytochemical Bioactive	Determined Concentrations
*Austrocedrus* *chilensis* (Ciprés de cordillera) Donoso-Fierro C et al. (2015) [173]	Heartwood	Yateinpure	cytotoxic effects on P3X murine myeloma cells	Yatein,	Effects shown to 12.5 µg/mL −25 µg/mL
*Gracilaria chilensis* (Pelillo) Torres-Estay V et al. (2025) [177]	Whole algae	Oily extract knownas Gracilex^®^	-Cytotoxic effects on prostate cancer cell lines (LNCaP and PC-3), reducing viability -Inhibition of migration, invasion, and tumor growth of injected PC-3 cells in xenograft models.	Attributed to its chemical composition, particularly its high gamma-tocopherol content	-IC50= 60 µg/mL -Three times per week for five weeks at a dose of 300 mg/kg/bw
*Kageneckia* *oblonga* (Bollén) Delporte C et al. (2002) [178]	Aerial parts	Global methanolic extract (GME)	Cytotoxicity against P-388 murine leukemia, A-549 human lung carcinoma, and HT-29 cell lines.	23,24-dihydrocucurbitacins	IC50 of GME = 2.5 μg/mL

## Data Availability

No new data were created or analyzed in this study.

## Abbreviations

The following abbreviations are used in this manuscript:

A-549	Human lung carcinoma cell line
ACHN	Human renal epithelial cancer cell line
AcOEt	Ethyl acetate
AGS	Human gastric adenocarcinoma cell line
AKT	Protein kinase B
Bax	Bcl-2-associated X protein
Bcl-2	B-cell lymphoma 2, protein
BE	*Boldus* extracts
BSB	N-benzylsecoboldine hydrochloride
C6	A glial cell line isolated from the rat with a glioma
CaCo-2	Immortalized human colorectal adenocarcinoma cell line
CCD841/CoN	Epithelial cell line isolated from the colon
c-fos	Fos proto-oncogene
COX-2	Cyclooxygenase 2
DCM	Dichloromethane
DNA	Deoxyribonucleic Acid
DU-145	Human prostate cancer cell line
EA.hy926	Endothelial somatic hybrid cell line
ERK	Extracellular Signal-Regulated Kinase
EtOH	Ethanol
F5	Fraction 5
G2	Gap 2 phase
G2/M	Gap 2/mitosis transition phase
GAE	Gallic acid equivalents
GES-1	Human gastric epithelial cell line
GRX	Immortalized murine hepatic stellate cell line
GSK-3β	Glycogen synthase kinase-3 beta
H_2_O_2_	Hydrogen peroxide
H460	Human lung carcinoma cell line
HaCaT	Human spontaneously immortal keratinocyte cell line
HBPs	Honeybee pollen
HEK-293	Human embryonic kidney 293 cells
HeLa	Epithelial cell line isolated from cervical carcinoma
HepG2	Human hepatocellular carcinoma cell line
Hex	Hexane
HPLC	High-Performance Liquid Chromatography
HSP70	Heat shock 70 protein
HT-22	Mouse neuronal immortalized cell line
HT-29	Human colon adenocarcinoma cell line
HTC 116	Human colorectal carcinoma cell line
HTR-8/SVneo	Human trophoblast cell line
Huh-7	Human hepatocarcinoma cell line
IARC	International agency for research on cancer
IC50	Half maximal inhibitory concentration
IκBα	Inhibitor kappa B alpha
IL-10	Interleukin 10
IL-4	Interleukin 4
IL-6	Interleukin 6
iNOS	Inducible nitric oxide synthase
Ki67	Proliferation marker protein Ki-67
LD25	25% of the maximal lethal dose
LD50	50% of the maximal lethal dose
LDH	Lactate dehydrogenase
LNCap	Human prostate cancer cell line
LTC	Leptocarpin
M14	Human melanoma cell line
MAPK	Mitogen-Activated Protein Kinase
MAPLC3α	Microtubule-associated protein light chain 3 alpha
MAPLC3β	Microtubule-associated protein light chain 3 beta
MCF-10F	Non-tumorigenic epithelial cell line from the human breast
MCF-7	Breast cancer cell line, estrogen receptor positive
MDA-MB-231	Breast cancer cell line, estrogen receptor negative
MEE	Maqui berry ethanol extract
MKN-45	Human gastric adenocarcinoma cell line
*mmp2*	Matrix metalloproteinase 2 gene
MnSOD	Superoxide dismutase
NBol	Nanoparticles of boldine
NCI-H1975	Human non-small cell lung cancer cell line
NFAT	Nuclear Factor of Activated T-cells
NF-κB	Nuclear factor kappa B
NO	Nitric oxide
P-388	Murine lymphocytic leukemia cell line
P3X	B lymphoblast cell line isolated from a plasmacytoma mouse
p53	Protein 53 (tumoral)
PC-3	Human prostate cancer cell line
PLGA	Poly lactide-co-glycolide, nanoparticles
PPARγ	Peroxisome proliferator-activated receptor gamma
QE	Quercetin equivalent, phalvoneoid
RAW-264.7	Murine macrophage cell line
SCC-4	Human squamous carcinoma cell line
SKHep-1	Human endothelial cell line, isolated from the liver adenocarcinoma patient.
SKOV3	Ovarian cancer cell line
SLs	Sesquiterpene lactones
SPF	Sun protection factor
STAT3	Signal transducer and activator of transcription 3
T84	Human colon adenocarcinoma cell line
TERT	Telomerase reverse transcriptase
TNF-α	Tumor necrosis factor Alpha
TopII	DNA topoisomerase II
U138-MG	Human glioblastoma cell line
U87-MG	Human glioblastoma cell line
UV	Ultraviolet
UVB	Ultraviolet B radiation
UVR	Ultraviolet radiation
WHO	World Health Organization
ZR-75-1	Breast cancer cell lines
